# Influence of the Physical State of Microencapsulated PCM on the Pressure Drop of Slurry in a Circular Channel

**DOI:** 10.3390/ma15196719

**Published:** 2022-09-27

**Authors:** Krzysztof Dutkowski, Marcin Kruzel, Dominika Kaczmarek, Bartłomiej Nalepa, Bartosz Zajączkowski, Jan Valíček, Marta Harničárová

**Affiliations:** 1 Faculty of Mechanical Engineering, Koszalin University of Technology, ul. Raclawicka 15-17, 75-620 Koszalin, Poland; 2 Department of Thermal Sciences, Wroclaw University of Science and Technology, 50-370 Wroclaw, Poland; 3 Department of Mechanical Engineering, Faculty of Technology, Institute of Technology and Business in České Budějovice, Okružní 10, 370 01 České Budějovice, Czech Republic or; 4 Department of Electrical Engineering, Automation and Informatics, Faculty of Engineering, Slovak, University of Agriculture in Nitra, Tr. A. Hlinku 2, 949 76 Nitra, Slovakia

**Keywords:** experimental investigation, pressure drop, microencapsulated PCM, slurry, phase change

## Abstract

Phase Change Material (PCM) is mainly used in thermal energy storage. The addition of small PCM particles to the working fluid circulating in the heat exchange systems allowed to increase the amount of transported energy thanks to the use of latent heat—the heat of phase change. Encapsulating PCM in microcapsules avoids the disadvantages of PCM emulsions and makes the resulting slurry an attractive heat energy carrier. The paper presents the effect of the aggregate state of PCM enclosed in microcapsules on the flow resistance of the slurry through a rectilinear tubular channel. The tests were carried out with the use of a tube with an internal diameter of 4 mm and a measuring section length of 400 mm. A slurry of 21.5 wt.% PCM microcapsules (MPCM) was used as the working fluid in distilled water. A slurry with temperatures of 18.4 °C (PCM encapsulated in a solid state), 26.1 °C (PCM is in a phase change), and 30.5 °C (PCM in a liquid state) flowed through the measuring section. The mass flow rate of the MPCM slurry reached 70 kg/h (Re_max_ = 2150). It was shown that the higher the Re number, the higher the value of the flow resistance, and the more clearly this value depended on the temperature of the slurry. Detailed analyses indicate that the observed changes were not the result of a change in the viscosity of the slurry, but its density depending on the state of the PCM. Significant changes in the density of the slurry in the range of the phase transition temperature are the result of significant changes in the volume of the microcapsule containing the phase change material in different aggregate states.

## 1. Introduction

Phase-change materials (PCMs) are now under the spotlight due to rapidly increasing interest in thermal storage systems. Their attractive ability to use latent heat for energy accumulation at almost on-demand above-zero temperatures opens many possibilities for applications [1,2,3]. PCM-enhanced working fluids tend to reduce power consumption, allowing the flattening of the curve during peak demand periods. A working fluid consists of a carrier fluid (usually water) and some form of PCM material as the dispersed phase. It uses the latent heat capacity of the PCM as well as the sensible heat capacity of the carrier fluid and the PCM to store or transfer thermal energy. For this reason, many researchers investigated the properties and application of PCMs to improve the efficiency of thermal systems [4,5,6]. The studies of working fluids with PCM are generally divided between PCM emulsions [7] and microencapsulated PCM slurries (MPCM or MPCM in short) [2,6], sometimes described as MPCMS (Microencapsulated Phase Change Material Slurry) or MPCS (Microencapsulated Phase Change Slurry) [4], where the capital S stands for slurry.

Today, microencapsulated PCMs are taking over as one of the most popular techniques to improve the efficiency in the use of resources for thermal energy storage [3]. Microencapsulation addresses leakage, phase separation, and volume change problems. It is a process by which very tiny particles or droplets are surrounded or coated with a polymer film. Microencapsulation was developed mainly by the pharmaceutical industries [8] and found its use in many functional PCM products, including thermal regulation building materials, clothing fabric, PCM heat transmittance emulsion, and infrared camouflage materials [9]. Microcapsules can handle PCMs as core materials with many advantages: decreasing PCMs tolerate volume change, preventing PCMs from contaminating the outside environment, and increasing the area of PCM heat transfer [9]. Several recent review articles [2,3,10] discuss the properties and applications of MPCM, focusing on the preparation methods, thermal properties, and potential applications of the resulting material. The authors explained the differences between different techniques (physical, chemical, and physical-chemical), highlighting advantages (e.g., low cost, control over the particle size) and the most common problems (e.g., tendency to agglomerate, short shelf life, poor mechanical performance). Although latent heat reduces after microencapsulation (since the addition of shell material reduces the proportion of PCM, and only PCM is used as an energy storage medium) [10], it was reported that MPCM shells do not have a large impact on the phase change temperature of pure paraffin [9]. On the contrary, [4] observed a significant enhancement in heat transfer due to direct surface contact between the encapsulated PCM and the heat transfer fluid, and at the same time pressure drop and viscosity increased substantially with an increasing volumetric concentration of the microcapsules. Additionally, the issue of the lower thermal conductivity of the shell can be improved with the proper selection and preparation of better coating materials, for example, metals such as silver [11].

This study focuses on the flow of MPCM slurry and related pressure drops. In principle, the turbulent flow of MPCM slurry is desirable for efficient heat exchange, but small hard spheres in particle-laden flow yield a greater pressure drop. On the other hand, laminar flow with a high mass fraction of MPCM particles provides a high energy transportation density, reduced pumping power, and reduced heat loss compared to the case of a system using a single-phase working fluid [12]. Multiple works have been published about the flow of microencapsulated PCM and its corresponding thermal and rheological effects. Wang et al. [13] investigated the heat transfer characteristics of MPCM slurry flow in a circular horizontal tube focusing on the thermal aspects of the flow. The authors proposed heat transfer correlations for laminar and intermediate flows (up to Re = 3500). Chen et al. [14] presented the comparison between pressure drops and flow velocities for pure water and 15.8 wt.% of MPCMS at 10 °C and 20 °C, revealing that at the same mean velocity, the pressure drop of water is lower than that of MPCMS. Additionally, the authors found that when the heat transfer rate is large enough, under the same heat transfer rate, the pumping power of MPCMS is much smaller than that of pure water, so less pumping power is needed for MPCMS for the same transported thermal energy [14]. This last observation was later confirmed by Delgado et al. [15]. Slightly more turbulent flows (3000 < Re < 6000) were studied by Taherian et al. [16]. The authors performed pressure drop measurements under turbulent flow conditions for six different MPCM samples at various concentrations and flow velocities ranging from 0.5 to 1.3 m/s. In principle, the pressure drop is larger in the MPCM slurry than in the water, although, for two samples, the pressure drop of the MPCM slurry was comparable to that of water, despite the higher viscosity of the slurry. The authors concluded that there may be a difference in the mechanical properties of the shells of these two MPCMs, causing a drag reduction effect that takes place on the inner walls of the tubes (i.e., the resultant relative surface roughness of the tube decreases) [17]. Similarly, Alvarado et al. [18] studied the pressure drop of methyl stearate MPCM slurries in the range of Reynolds numbers 3600–11,300 and mass fractions between 5.9–15.2% as higher than water. This indicates that the effective surface roughness for such MPCMs is increased, and thereby the pressure drop raises as well.

In view of the trade-offs between mass flows and heat transfer efficiency, it is important for the system design to define the optimal flow regime.

This work is part of our broader investigation of the thermodynamic and rheological properties of various MPCM suspensions. Previous research included dynamic viscosity [19,20,21,22], density [23], the specific heat and latent heat of fusion [24,25], and apparent thermal conductivity [26]. Here, we determine the pressure drop for a simple straight-pipe hydraulic system filled with MPCM slurry. The (unknown from the literature) detailed experimental research was conducted for three different thermodynamic states of a PCM material inside microcapsules: solid, during the solid–liquid transition, and liquid. The influence of the PCM state of aggregation on the value of the slurry flow resistance was demonstrated.

Detailed analyses indicate that the observed changes in the pressure drop were not the result of a change in the viscosity of the slurry, but its density depending on the state of the PCM. It was found that the significant changes in the density of the suspension in the range of the phase change temperature are the result of significant changes in the volume of the microcapsule containing the phase change material in a different state of aggregation.

## 2. Test Stand

### 2.1. Working Fluid

**Figure 1:**
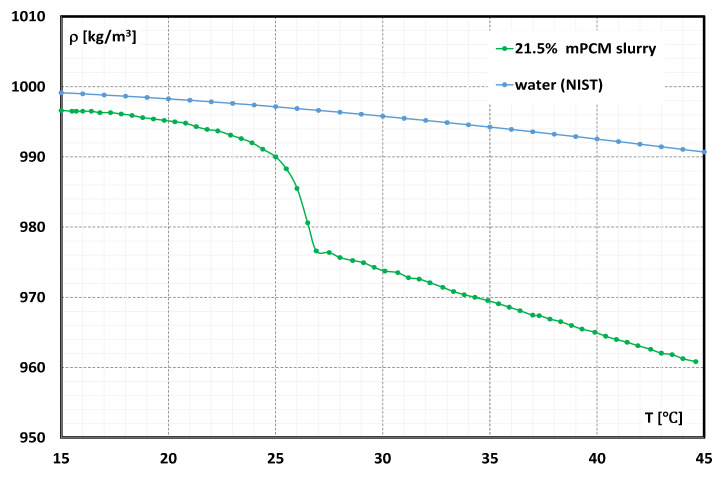
The dependence of slurry density (21.5% MPCM) on temperature.

**Figure 2:**
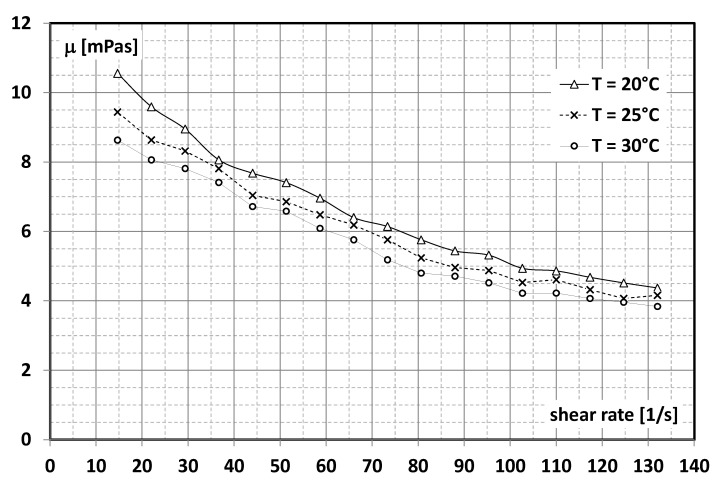
Dependence of slurry’s viscosity (21.5% MPCM) on the shear rate and temperature.

A slurry of microencapsulated PCM with the trade name MICRONAL^®^ 5428, a product of Microtek Labs (Moraine, OH, USA), was used for the tests. According to the manufacturer’s data [27], the product (concentrate) consisted of 57% water as the base liquid and 43% ± 1% wt. microcapsules filled with paraffin wax. The melting point of the paraffin used was 28 °C ± 1 °C. Microcapsules 1–5 µm in size are made of polymethylmethacrylate polymer. The concentrate has a viscosity that makes it practically impossible to use it directly in thermal circuits. A suspension consisting of 50% concentrate and 50% distilled water was prepared for the tests. Ultimately, the content of MPCM in the slurry constituting the working liquid used for the tests was 21.5%. The prepared sample was subjected to basic tests, the purpose of which was to determine its physical properties, including density and viscosity. The density of the suspension used for the tests (Figure 1) was determined by the method described in detail by the authors in [23], and the viscosity (Figure 2) by the method described in [20,21,22].

It is noticed from Figure 1 that when the MPCM slurry contains paraffin in the form of a solid or in the form of a liquid, the change in temperature causes slight changes in the density of the slurry. During the phase change, the volume of the microcapsules loaded with the paraffin wax undergoes significant changes, which is reflected in the rapidly changing value of the slurry’s density.

From Figure 2, it is noted that the viscosity of the slurry depends on the shear rate. Along with the increase in the shear rate, the value of the dynamic viscosity coefficient of the MPCM slurry decreases. The temperature of the slurry also influences the viscosity coefficient of the slurry. For the same shear rate values, the higher the temperature of the slurry, the lower its viscosity becomes.

### 2.2. Experimental Facility

**Figure 3:**
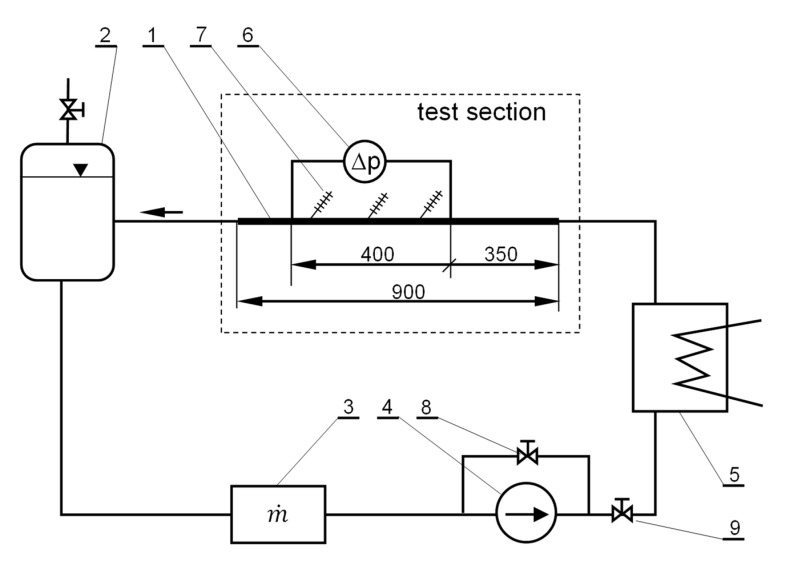
Diagram of the test stand; 1—test section, 2—working liquid tank, 3—flow meter, 4—working medium pump, 5—heat exchanger, 6—differential pressure sensor, 7—thermoelectric thermometer, 8—bypass control valve, 9—main control valve.

The test stand, the diagram of which is shown in Figure 3, consisted of successive elements constituting a closed cycle for the flowing working fluid.

The main element of the installation was the test section consisting of a straight section of a pipe with a constant internal diameter (no. 1 in Figure 3). The tube installed at the stand was made of a “hard” copper straight pipe. The tube had a total length of 900 mm and an internal diameter of 4 mm. Two impulse openings are made in the tube, enabling the measurement of the local value of the static pressure of the flowing liquid. The pulse openings were arranged such that the entire tube was divided into three sections. The first section, 350 mm long, was a hydraulic inrun zone. The boundary layer was formed in this section, and the flowing liquid obtained a stable flow structure. The second section, 400 mm long, was the actual measuring section, along which the pressure drop was measured. The third section, 150 mm long, is an outlet zone that allows the undisturbed outflow of liquid from the test section. In the place where there were impulse openings, stub pipes were attached to enable the connection of a pressure drop measuring device. Three thermometers for measuring the temperature of the flowing liquid were evenly placed on the outer surface of the tube over a specific 400 mm test length. The entire section was thermally insulated with a rubber cover. The working fluid leaving the test section flowed into the tank (2). From there, the liquid flew through the miniature gear pump (4) and through the flow meter (3). A throttle valve installed downstream of the pump (9) and a valve installed on the bypass (8) allowed for a smooth adjustment of the liquid flow rate. The liquid of the known flow rate was directed to the plate heat exchanger (5). Intermediate liquid with regulated temperature, flowing through the heat exchanger, made it possible to precisely control the temperature of the working liquid (MPCM slurry) flowing to the test section of the station.

### 2.3. Research Procedure and Scope of the Experiment

The MPCM slurry in the working liquid tank was directed to the flow meter to determine the amount of working fluid flowing through the test section. A mass flow meter based on the Coriolis effect—Promass 80, manufactured by Endress-Hauser (Wien, Austria) was used for the measurements. The measuring range of the flow meter was 0 ÷ 200 kg/h, and the accuracy of the mass flow rate measurement is ±0.2% of the measured value. Then, the slurry, with a known mass flow rate, flowed through the pump and the control valve section. Using the control valves (main and on the by-belt—no. 8 and 9 in Figure 3), the flow rate of the slurry was changed. The maximum value of the mass flow rate of the slurry during the tests reached 70 kg/h. Subsequently, the slurry flowed to the plate heat exchanger. Its use made it possible to regulate the temperature of the slurry flowing into the research section. The value of the temperature of the slurry taken for further analyses was the average of the readings of three thermometers placed evenly on the surface of the tube over a section whose length was 400 mm. K-type thermocouples, individually created and marked in the temperature range of 10–60 °C, were used as thermometers. The accuracy of the temperature measurement obtained after marking the thermometers was ±0.2 °C. In addition, the rubber cover limited heat loss to the environment, ensuring a constant temperature of the slurry along the length of the research section. Heat losses were negligible due to the fact that the temperature of the working fluid during the tests (18.4 °C, 26.1 °C, and 30.5 °C) was close to the ambient temperature. The measurement of the pressure drop as the slurry flows through a tube with an internal diameter of 4 mm and a length of 400 mm was carried out with a Deltabar S PMD75 piezoresistive differential pressure sensor manufactured by Endress-Hauser (Wien, Austria). The measuring range of the device, created in the 0.075 class, was 0 ÷ 50 kPa. Hence, the maximum error of the pressure drop measurement was ±37.5 Pa. All measurements were taken in a steady state.

## 3. Experimental Data

**Figure 4:**
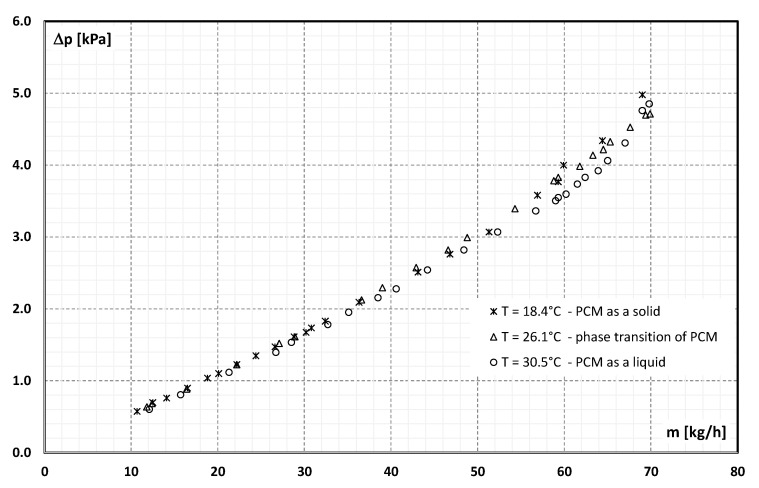
The dependency of pressure drop of the slurry (21.5% MPCM) in a tube 400 mm long and 4 mm d_i_ on the mass flow rate and temperature.

Figure 4 shows the results of the measurement of the static pressure drop of the suspension over a length of 400 mm obtained from the experimental tests. Measurements were taken in three measurement series with different temperatures of the suspension. From Figure 1, it is noted that the slurry temperature of 18.4 °C corresponds to the situation where the PCM encapsulated in the microcapsule is in the solid state, 26.1 °C—the PCM is in a phase change, and 30.5 °C—the PCM is in the liquid state.

It can be seen from Figure 4 that with the increase in the flow rate, the value of the pressure drop of the slurry increases. The nature of the changes does not differ from the classical theories of fluid mechanics. There is also no noticeable influence of the aggregate state of the PCM on the pressure drop values obtained. 

**Figure 5:**
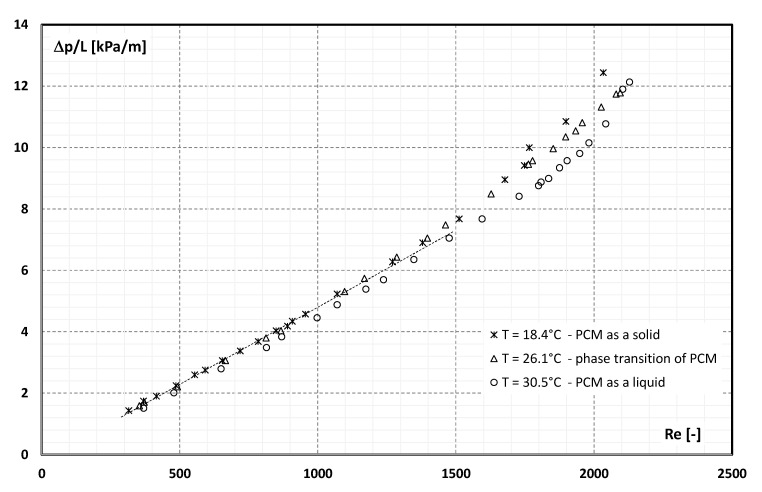
The slurry flow resistance (21.5% MPCM) as a function of Reynolds number and temperature.

Figure 5 shows the effect of the MPCM slurry flow velocity on its flow resistance. The flow rate of the slurry was expressed in a dimensionless form as a Reynolds number. The Re of the slurry was determined according to Formula (1):

(1) $$Re=\frac{w\cdot d}{\nu }$$

where: *w*—the average flow velocity of the slurry [m/s], *d*—the inner diameter [m] of the channel used during the research and *ν*—the kinematic viscosity coefficient [m^2^/s] **aqueous solution of mPCM**. Assuming that the velocity of the slurry flow can be determined from the formula:

(2) $$\dot{m}=\rho \cdot Q=\rho \cdot w\cdot A=\rho \cdot w\cdot \frac{\pi \cdot d^{2}}{4}$$

where: $\dot{m}$—slurry mass flow rate [kg/s], *ρ*—slurry density [kg/m^3^], *Q*—slurry volumetric flow rate [m^3^/s] **i *A***—**cross-sectional area of the channel [m^2^]**.

The kinematic viscosity coefficient ν as a function of the dynamic viscosity coefficient µ [mPa⋅s], as:

(3) $$\nu =\frac{\mu }{\rho }$$

Determining the velocity of the slurry from Formula (2) and assuming the kinematic viscosity coefficient ν according to Equation (3), eventually, Formula (1) takes the form

(4) $$Re=\frac{4\dot{m}}{\pi \cdot d\cdot \mu }$$

In order to determine the Reynolds number, it is necessary to know the value of the dynamic viscosity coefficient, which is presented in Figure 2 as a function of the shear rate $\dot{\gamma }$ [1/s]. The shear rate $\dot{\gamma }$ dependency on the mass flow rate was determined from the relationship applicable to the fluid flow in the pipe channels:

(5) $$\dot{\gamma }=\frac{32Q}{\pi d^{3}}=\frac{32}{\pi d^{3}}\frac{\dot{m}}{\rho }$$

Using Formula (5), it was possible to determine, for each value of the mass flow rate $\dot{m}$ of the suspension, the corresponding value of the shear rate $\dot{\gamma }$, and hence from Figure 2, the dynamic viscosity coefficient of the slurry $\dot{\gamma }$. The value of the dynamic viscosity coefficient was used to calculate the Reynolds number *Re*.

Figure 5 shows the characteristics of the flow resistance of the MPCM slurry on the *Re* number for three cases: when PCM enclosed in a microcapsule is in the form of a solid (T = 18.4 °C), PCM undergoes a phase change (T = 26.1 °C) and has completely undergone melt (T = 30.5 °C).

It can be noted from Figure 5 that the scope of the experiment (*Re* range) coincides with the range considered as laminar flow in classical fluid mechanics. It is observed that as the value of *Re* increases, the flow resistance increases. In the range of *Re* <1500, the flow resistances are only a function of *Re* and do not depend on the temperature of the slurry. The flow resistances start to differ when the value of *Re* = 1500 is exceeded. As it can be seen in Figure 2, at high values of the shear rate, i.e., at high *Re*, the differences in the values of the dynamic viscosity coefficient as a function of temperature are insignificant. The effect of a clear difference in the flow resistance, for the same (high) *Re* number, should be explained by a clear change in the density of the slurry (Figure 1) resulting from the aggregate state of the PCM. The highest flow resistance occurs for the slurry in which PCM is in the form of a solid. The melting process of PCM causes the slurry flow resistance to be lower. Additionally, it is noted that after exceeding the value of *Re* = 1500, the experimental points no longer appear along a straight line. This may indicate an earlier change in the nature of the fluid movement and a deviation from the laminar flow of the slurry. This fact is confirmed, inter alia, in the works [28,29,30]. In the world literature, no detailed experimental data on MPCM slurry have been found, in which the obtained results would take into account the assessment of the impact of the aggregate state of the PCM material on the results of the research. Therefore, it should be hypothesized that in MPCM slurries, the turbulent flow resistance depends on the aggregate state of the material enclosed in the microcapsules. This hypothesis requires further detailed research.

## 4. Summary and Conclusions

Detailed studies of the flow resistance of the microencapsulated PCM slurry during the flow through a pipe channel with an internal diameter of 4 mm were carried out. An aqueous slurry with a content of 21.5% wt. in the microcapsules was previously tested, during which its viscosity (as a function of temperature and shear rate) and density (as a function of temperature) were determined. The flow resistance tests were carried out for three different values of the slurry temperature: 18.4 °C—PCM enclosed in a microcapsule is in a solid state; 30.5 °C—PCM is in the liquid state and 26.1 °C—PCM is undergoing a phase change. The tests were carried out in the range of mass flow rate from 10 kg/h to 70 kg/h, which corresponded to the range of *Re* = 300 ÷ 2200. The detailed physical properties of the MPCM slurry used in the tests allowed to determine the influence of the MPCM aggregate state on the flow resistance. It was found that:

- pressure drop during the flow of 21.5 wt.% of MPCM water slurry in a pipe channel with an internal diameter of 4 mm and a length of 400 mm varied from 0.5 kPa to 5 kPa,
- the flow resistance of the slurry depended on the Reynolds number and, for *Re* >1500, on the aggregate state of the PCM in the microcapsules,
- the more the *Re* number was higher than the value of 1500, the more the slurry flow resistance depended on the aggregate state of the PCM,
- the highest flow resistance was recorded when the PCM in microcapsules was in the form of a solid, and the lowest when the PCM was in the form of a liquid,
- the flow resistance in the range *Re* = 300 ÷ 1500 changed from 1.5 kPa/m to about 7 kPa/m and created a rectilinear characteristic, which is consistent with the theory of laminar fluid flow in pipe channels,
- for *Re* = 1500, the characteristic of flow resistance begins to diverge from a linear trend, taking the shape of a different parabola for each of the PCM aggregate states,
- The hypothesis about the significant influence of the microcapsule state of aggregation on the resistance of the turbulent flow of MPCM slurries requires further detailed verification tests.

## Data Availability

Not applicable.
